# Genome-Wide Analysis of Androgen Receptor Targets Reveals COUP-TF1 as a Novel Player in Human Prostate Cancer

**DOI:** 10.1371/journal.pone.0046467

**Published:** 2012-10-04

**Authors:** Ruth Perets, Tommy Kaplan, Ilan Stein, Guy Hidas, Shay Tayeb, Eti Avraham, Yinon Ben-Neriah, Itamar Simon, Eli Pikarsky

**Affiliations:** 1 Department of Pathology and Lautenberg center for immunology, IMRIC, The Hebrew University-Hadassah Medical School, Jerusalem, Israel; 2 Department of Urology, The Hebrew University-Hadassah Medical School, Jerusalem, Israel; 3 Department of Microbiology and Molecular Genetics, Institute for Medical Research Israel Canada (IMRIC), The Hebrew University-Hadassah Medical School, Jerusalem, Israel; 4 Department of Molecular and Cell Biology, California Institute of Quantitative Biosciences, University of California, Berkeley, California, United States of America; 5 School of Computer Science and Engineering, The Hebrew University of Jerusalem, Jerusalem, Israel; 6 Division of Oncology, Rambam Health Care Campus, Haifa, Israel; Clermont Université, France

## Abstract

Androgen activity plays a key role in prostate cancer progression. Androgen receptor (AR) is the main mediator of androgen activity in the prostate, through its ability to act as a transcription mediator. Here we performed a genome-wide analysis of human AR binding to promoters in the presence of an agonist or antagonist in an androgen dependent prostate cancer cell line. Many of the AR bound promoters are bound in all examined conditions while others are bound only in the presence of an agonist or antagonist. Several motifs are enriched in AR bound promoters, including the AR Response Element (ARE) half-site and recognition elements for the transcription factors OCT1 and SOX9. This suggests that these 3 factors could define a module of co-operating transcription factors in the prostate. Interestingly, AR bound promoters are preferentially located in AT rich genomic regions. Analysis of mRNA expression identified chicken ovalbumin upstream promoter-transcription factor 1 (COUP-TF1) as a direct AR target gene that is downregulated upon binding by the agonist liganded AR. COUP-TF1 immunostaining revealed nucleolar localization of COUP-TF1 in epithelium of human androgen dependent prostate cancer, but not in adjacent benign prostate epithelium. Stromal cells both in human and mouse prostate show nuclear COUP-TF1 staining. We further show that there is an inverse correlation between COUP-TF1 expression in prostate stromal cells and the rising levels of androgen with advancing puberty. This study extends the pool of recognized putative AR targets and identifies a negatively regulated target of AR – COUP-TF1 – which could possibly play a role in human prostate cancer.

## Introduction

Prostate cancer is the most common non-skin cancer in males in the US, with an estimated number of 217,730 new cases in the US in 2010 [1]. Androgen deprivation therapy is currently the mainstay for advanced prostate cancer treatment. Androgen deprivation can be achieved through androgen depletion (e.g treatment with GnRH agonists) sometimes in combination with androgen antagonists such as flutamide and bicalutamide [2]–[4].

Androgen's effect on normal and malignant prostate cells is mediated through its ability to enter cells and bind its receptor – the AR. In the absence of a ligand the AR is located in the cytoplasm in a complex with heat-shock proteins (HSP) and co-chaperones [5]–[7]. Upon androgen binding the AR undergoes structural rearrangement which results in dissociation of HSP, exposure of its nuclear localization signal and translocation into the nucleus. Nuclear AR binds DNA, recruits co-activators and facilitates transcription of target genes. The transcription of target genes is considered to be the major means through which the AR affects the cells. Ligand bound steroid receptors were canonically believed to bind a consensus sequence in DNA that is made up of two hexameric half-sites of the consensus sequence 5′-TGTTCT-3′, arranged as inverted repeats, separated by three nucleotides [8]–[15]; yet this dogma was recently contended with regard to the AR. It was recently suggested, as supported by our data, that the half site is sufficient for AR binding to DNA in the presence of androgen [16]–[18].

In the presence of an AR antagonist, such as flutamide, the AR transcriptional complex still forms, yet transcription of well known AR target genes does not occur possibly via the recruitment of co-repressors. For example, upon addition of the antagonist bicalutamide, AR shifts into the nucleus, binds the promoter of its well known target gene PSA and recruits co-repressors such as SMRT and NCoR [19], [20]. The formation of the antagonist bound AR transcriptional complex was widely studied on single promoters [19]–[21]. However, the genome-wide promoter occupancy of antagonist bound AR was never studied before. We hypothesized that in androgen dependent prostate cancer cells antagonist bound AR binds a unique set of target genes, that might differ from the target genes of agonist bound AR.

We have utilized genome-wide location analysis of AR in the presence of agonist, antagonist or no ligand to study the differences and similarities between AR target genes in those conditions. We have seen several promoters that are constitutively bound in the presence of an agonist and antagonist, as well as promoters that are bound only in the presence of either one. We further characterize one novel AR negatively regulated target gene COUP-TF1, which promoter is bound only in the presence of the antagonist.

## Results

### Androgen Receptor target genes in human prostate cancer cells

LAPC4 prostate cancer cells express wild type AR [22], reflecting the AR status of most androgen dependent prostate cancers. In some prostate cancer cell lines, certain AR antagonists can serve as agonists, probably due to the presence of a mutant AR [23]–[27]. Thus, we first tested the effect of androgen, or AR antagonist on growth of LAPC4 cells in vitro compared with cells treated with vehicle alone. LAPC4 cells proliferated in the presence of androgen, but not in the presence of an antagonist. When combined together flutamide antagonizes the proliferative effect of androgen (figure S1). These results confirm the androgen dependence of LAPC4 cells, show that flutamide serves as an antagonist of AR's proliferative effect and rule out the possibility that flutamide can serve as a functional AR agonist in this cell line.

To identify the direct target genes of AR in prostate cancer cells in the presence of an AR agonist, AR antagonist, or in the absence of both, LAPC4 cells were first androgen ablated for three days. The cells were then incubated with vehicle alone, 10 nM R1881 or 40 **µ**M flutamide for 16 hours. The lengths of activation and R1881 concentration were chosen according to the time of maximal AR recruitment to its best studied target gene PSA [28]. Chromatin immuno-precipitation (ChIP) of AR bound chromatin was performed as described in materials and methods. The immuno-precipitated fraction and a sample of the input DNA were hybridized to a microarray representing 19,000 human gene promoters. Binding data from three ChIP-chip experiments was analyzed and probes that were bound with p-value<0.001 were considered as AR bound promoters (ARBs).

**Figure 1:**
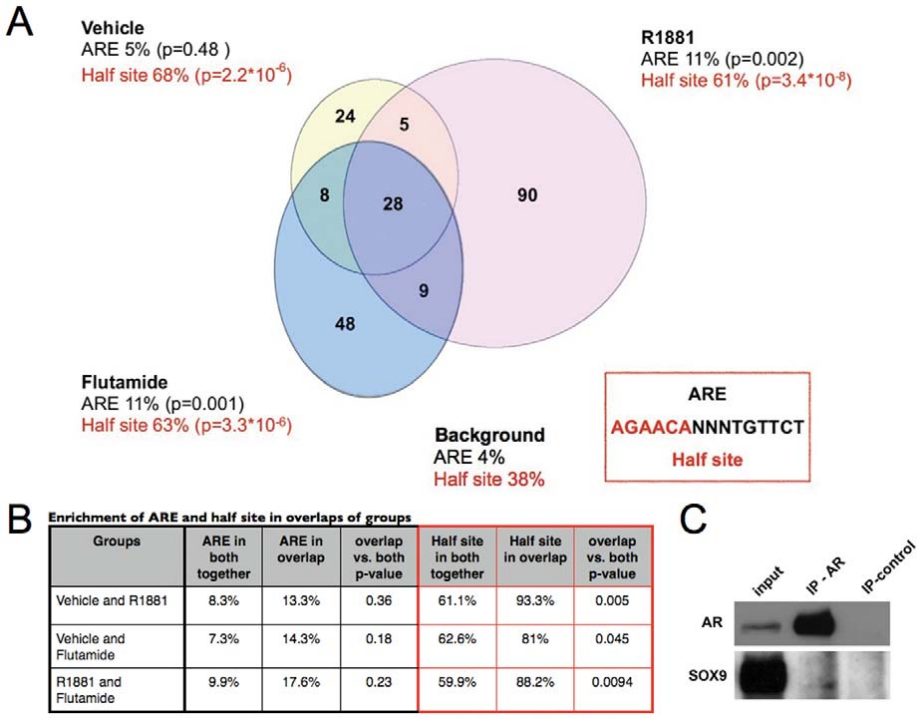
ChIP on chip analysis of AR bound promoters in LAPC4 cells. LACP4 cells were androgen deprived for 72 hours and then treated with vehicle (ethanol), a synthetic androgen (R1881) or the AR antagonist flutamide. Cells were fixed 16 hours after treatment and ChIP on chip analysis was performed to identify AR bound promoters. A. Number of AR bound promoters in each treatment group, and overlap between groups are presented in the Venn diagram. In each treatment group the sequence of the promoter's probe in the array was analyzed for presence of the classical AR response element (ARE) or the half site. Shown are frequencies of sequences containing the ARE or half site found in each treatment group and the frequency in all of the probes on the array (background). A p-value of enrichment was calculated based on group frequency compared to the background of the array using standard student's t-test. Inset box shows the classical AR response element sequence. B. Table showing the enrichment of ARE and half site in overlaps between groups. For each pair of experimental conditions the frequency of ARE and half site is calculated in overlapping promoters and in all promoters of both groups. P-value of enrichment in overlapping promoters compared to all promoters is calculated using hypergeometric distribution. C. Co-immunoprecipitation of AR and SOX9 in LAPC4 cells.

An analysis of AR target genes with vehicle alone, R1881 and flutamide revealed three groups of ARBs. There is some overlap between target genes in those three conditions, as well as ARBs specific to each condition (figure 1a and table S2). The lists of target genes revealed some genes that were known to be regulated by AR such as HOXB13 [29], [30]. Some well-known AR target genes such as PSA were not retrieved in these arrays despite the fact that gene specific ChIP indicated that it is preferentially bound by AR (figure S2a). This implies a certain rate of false negative findings. However as the full set of targets is unknown the false negative rate cannot be estimated.

**Figure 2:**
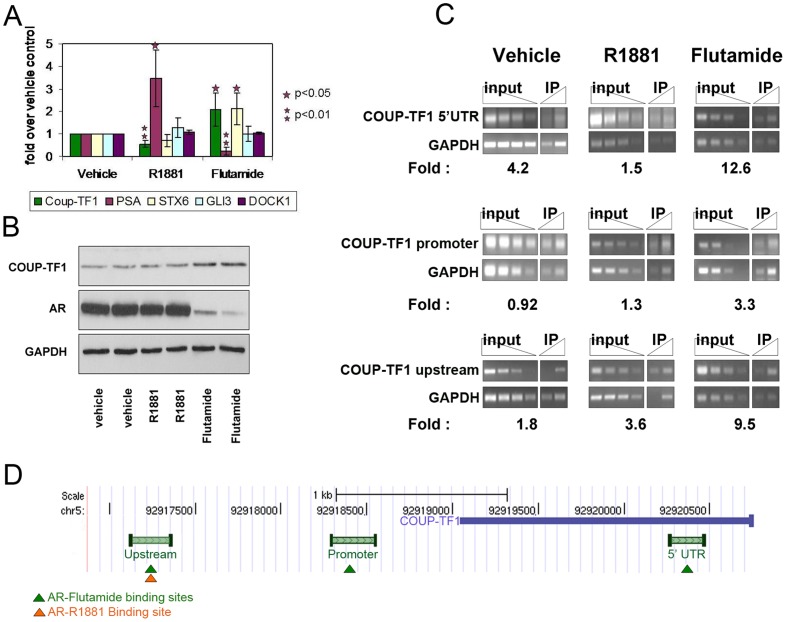
COUP-TF1 is a direct negatively regulated target of AR. A. LAPC4 cells were androgen deprived for 72 hours and then treated with a synthetic androgen (R1881), an AR antagonist (flutamide) or vehicle (ethanol) for 24 hours. Real-time PCR was used to quantify mRNA levels of the indicated genes. Each expression level was adjusted to GAPDH mRNA level and shown as fold change from vehicle treated control. Each bar represents at least four experiments each done in triplicates. P-value was calculated using student's t-test, compared to vehicle treatment. B. Western blot analysis showing COUP-TF1 and AR expression levels following treatment with R1881 or Flutamide for 48 hours. Each treatment group was performed and shown in duplicates. C. Analysis of AR binding to the COUP-TF1 genomic locus. LAPC4 cells were androgen deprived for 72 hours and then treated with a synthetic androgen (R1881), an AR antagonist (flutamide) or vehicle (ethanol) for 16 hours. ChIP with an anti AR antibody was performed. PCR for the indicated areas surrounding the COUP-TF1 transcription start site compared to non-bound gene are presented for 3 fold dilutions of input and immunoprecipitated fraction. Enrichment of binding to each region compared to a non-bound promoter (GAPDH) is quantified below each image. PCR for the 5′ UTR of COUP-TF1 is presented in uppermost panel (COUP-TF1 5′UTR), binding to the COUP-TF1 promoter is presented in the middle panel and binding to a region upstream to the promoter (COUP-TF1 upstream) is presented in the lowermost panel. D. Schematic representation of AR binding sites in the genomic locus surrounding coup-tf1 TSS, shown in figure C. Green bars represent areas analyzed for AR binding. Genomic locations of AR binding in the presence of flutamide and R1881 are marked by green and orange triangles respectively.

Genome-wide location analysis results were validated using gene specific chromatin IP for eight of the ARBs in all three treatments (see figure S2 and figure 2c). Chromatin IP was performed as described in materials and methods and PCR was used to quantify the amount of a specific DNA fragment in the precipitated fraction. Quantification of enrichment was done in a computational unbiased method. We used three fold enrichment as our binding cutoff (based on PSA-promoter binding, see figure S2a). We have validated binding for eight genomic locations, each in the presence of vehicle, agonist or antagonist. Out of the 24 conditions tested 18 were shown to be bound by AR in the array. Out of those, 16 were bound also by gene specific IP. Therefore we concluded that in 16/18 (89%) promoter-conditions tested, the gene specific data confirmed the array data, indicating a false positive rate of 11%.

Our experiments revealed constitutive binding of AR to the PSA promoter in the presence of vehicle, R1881 or flutamide (figure S2a), as expected [20]. Constitutive AR binding was validated for several other genes such as the novel ARBs *sox5* (figure S2b), *dock1* (figure S2c) and *slitrk3* (figure S2e). *IL1R2* is a novel ARB, bound in the presence of either agonist or antagonist, but not without a ligand (figures S2f). *B3gnt5* promoter is bound by the AR only in the presence of an agonist (figure S2d).

AR target genes are evenly distributed along the different chromosomes for all ligands tested, as analyzed by Webgestalt [31](figure S3).

Gene ontology annotation (GO) analysis was performed to find functional groups that are enriched within ARBs. In all ligand settings examined, despite great variation in target genes, the enriched categories, were those categories involved in DNA binding and transcription activity (table 1).

**Table 1:** Over represented Gene Ontology Categories.

	Observed	Expected	p-value
**Vehicle**			
DNA binding	11	4.9	0.00569
motor activity	3	0.26	0.00222
**R1881**			
transcription regulatory activity	17	8.77	0.00478
sequnce-specific DNA binding	9	3.42	0.00665
ligand-gated ion channel activity	4	0.82	0.00892
interferon-alpha/beta receptor binding	2	0.09	0.00347
N-methyltransferase activity	2	0.13	0.00733
**Flutamide**			
transcription factor activity	9	3.38	0.00505
sequence specific DNA biniding	7	1.83	0.00197
interleukin receptor activity	2	0.14	0.00795
interleukin-1 receptor activity	2	0.04	0.00059
interleukin-1 binding	2	0.05	0.00101

### ARE half site is prevalent in AR binding sites

We looked for the prevalence of the canonical androgen recognition element in the ARB set we identified, compared with all 18,051 probes on the array. We allowed for up to two mismatches in the 15 bp androgen response element (ARE) sequence. The ARE was found in 4% of all probes on the array. When scanning for ARE in the three lists of ARBs there was only mild enrichment of ARE compared to the background in the R1881 and flutamide groups (figure 1a). When scanning for ARE in the promoters that were bound in two of the conditions, compared to its prevalence in the promoters of both groups, there was no further enrichment (figure 1b). Similar results were described by others, both in AR bound promoters and AR bound enhancers [16]–[18]. Thus, our results support the notion that the dogmatic canonical ARE site does not, on its own, play a key role in AR recruitment.

Next, we asked whether the ARE half site (5′-AGAACA-3′) is enriched in any of the groups, without mismatches. The half-site was searched in ARBs compared with the array's background. The half-site ARE was found to be highly enriched in all three groups of ARBs (figure 1a). Hence the previously reported half site that is prevalent in ARBs in the presence of an agonist [16]–[18] is also enriched in the presence of an antagonist. The half site is further enriched in the promoters that are bound in two conditions, compared to its prevalence in both groups together (figure 1b). Therefore in the conditions we examined the half site is a universal recognition element for the androgen receptor, irrespective of the ligand.

### SOX9 and OCT1 are putative AR co-factors

A sequence analysis of ARBs was used to reveal AR co-transcription factors that could be commonly associated with it. In order to look for recognition elements of known transcription factors we used CIS [32] to scan the ARBs for previously defined recognition elements of known transcription factors. The enriched elements in each group of ARBs can be seen in Table 2. Among those elements, some, such as the OCT and the forkhead families of transcription factors, were previously reported to be involved in AR activity [17].

**Table 2:** Over represented Transfac motifs.

	vehicle		R1881		Flutamide	
Motif	Abundance	p-value	Abundance	p-value	Abundance	p-value
V_BRACH_01	17%	0.000764	10%	0.018684	20%	0.000001
V_SOX9_B1	18%	0.000179	11%	0.003374	15%	0.000285
V_OCT1_Q5_01	13%	0.009976	14%	0.000051	12%	0.009393
V_OCT1_B	15%	0.002924	12%	0.001297	13%	0.003207
V_FOXP1_01	17%	0.000764	16%	0.000004	11%	0.024955
V_MRF2_01	13%	0.009976	7%	0.141088	17%	0.000075
V_DMRT5_01	15%	0.002924	12%	0.001297	14%	0.000999
V_COREBINDINGFACTOR_Q6	10%	0.080054	13%	0.000164	13%	0.003264
V_OCT1_Q6	15%	0.002924	8%	0.077669	13%	0.003207
V_SRF_Q4	17%	0.000764	11%	0.003374	12%	0.009393
V_ICSBP_Q6	12%	0.030081	9%	0.039547	15%	0.000285
V_OCT_Q6	12%	0.030081	10%	0.008216	12%	0.009393
V_HNF3_Q6	13%	0.009976	11%	0.003374	12%	0.009393
V_OCT4_02	13%	0.009976	9%	0.039547	14%	0.000999
V_SOX5_01	12%	0.030081	9%	0.039547	11%	0.024955
V_FOXP3_Q4	13%	0.009976	4%	0.672383	11%	0.024955
V_TBP_01	10%	0.079708	13%	0.000474	8%	0.128745
V_IRF_Q6	10%	0.079363	7%	0.236353	14%	0.000999
V_SRF_C	10%	0.079363	10%	0.008216	13%	0.003207
V_FOXJ2_01	10%	0.079363	9%	0.039547	12%	0.009393

We found SOX9 element to be significantly enriched in ARBs in all three conditions tested. Specifically in the flutamide treated group of ARBs SOX9 recognition element was found in 15% of ARBs (p = 0.0003). Interestingly, SOX9 was recently described as active in prostate carcinogenesis and embryogenesis [33], [34] [35] and activated in response to androgen treatment in early prostate development [34]. Accordingly SOX9 and AR co-immunoprecipitate in LAPC4 cells (figure 1c). Taken together, Our data suggests SOX9 as a putative AR co-factor.

We further wished to look for novel motifs that were enriched in ARBs through *de novo* motif search. Weeder [36], an enumerative *de novo* motif search algorithm, revealed the motif GCAAATCA to be significantly enriched in the agonist bound group, and further analysis revealed it to be enriched in all ARB groups (table 3 upper part). This sequence overlaps the canonical OCT1 recognition element ATGCAAAT. The canonical OCT1 recognition element is also prevalent in our list of ARBs though not as significantly as GCAAATCA (table 3 lower part).

**Table 3:** OCT1 canonical and non-canonical motifs - abundance in ARBs and p-value of.

Non-canonical OCT1 – GCAAATCA		
	Abundance	p-value
Vehicle	10.17%	0.034
R1881	16.79%	1.00E-10
Flutamide	13.25%	1.00E-14
background	4.59%	

Canonical OCT1 - ATGCAAAT		
	Abundance	p-value
Vehicle	15.25%	0.02
R1881	10.69%	0.14
Flutamide	15.66%	0.006
Background	8.08%	

### ARBs are located at AT-rich genomic regions

In order to further characterize the ARBs we calculated the GC content of all ARBs, and compared it to the array's background GC content. Surprisingly we found that the ARBs are highly and significantly AT rich. The GC content of the entire array is 54.3%, while the GC content of the agonist and antagonist ARBs are 46.4% (p = 1.6*10^−13^) and 48.5% (p = 3.4*10^−5^), respectively. In order to confirm that this phenomenon is not a selection bias of the array, we compared it to the GC content of MLL1 bound probes that were published using the same array [37]. MLL1 bound probes contained 56.1% GC, similar to the array's background. To further validate this result we calculated the GC content of previously published AR bound promoters. In the Massie *et al.* data set that analyzed androgen bound promoters in the presence of androgen [16] we found a GC content of 52.6% as compared to 53.6% in the entire array (p = 0.0004). In order to determine that this is not a general phenomenon of transcription factors we examined the GC content of p53 binding sites in response to irradiation [38]. The GC content of P53 binding sites is 55.7% in comparison to 52.5% for the background. Therefore, we conclude that AR tends to associate with AT rich promoters.

### AR binding to promoter does not suffice for androgen regulation of adjacent genes

To assess the relevance of AR binding to transcription we measured the mRNA expression of several ARB genes under the 3 different androgen treatments where they were found to bind, using real time PCR. As expected, some ARBs showed increased expression upon androgen treatment and decreased expression upon addition of the antagonist flutamide. However, other ARBs such as DOCK1 and GLI3 did not display an androgenic response under the test conditions (figure 2a). COUP-TF1, which was suggested by our genome-wide analysis to be bound by the AR in several locations surrounding the transcription start site (TSS), is negatively regulated by androgen (figure 2a).

Syntaxin 6 (STX6) is a vesicle transporter protein that was recently shown to be regulated by p53 and required for cancer cell adhesion and survival [39]. STX6 was revealed by our genome-wide location analysis to be bound by AR in the presence of either R1881 or flutamide. STX6 mRNA levels were slightly downregulated by R1881, although this did not reach statistical significance. However, STX6 was significantly upregulated by flutamide (figure 2a).

As expected, the well known AR target PSA is upregulated by androgen and down regulated by flutamide.

Thus, AR binding to promoter sites can be associated with either upregulation, downregulation or no change of transcription.

### COUP-TF1 regulation by AR agonists and antagonists

We further wanted to ask whether flutamide bound AR has a functional transcriptional role. To answer this question we chose to focus on one flutamide activated promoter – the promoter of Chicken Ovalbumin Upstream Promoter – Transcription Factor 1 (COUP-TF1). Coup-TF1 is an orphan nuclear receptor which acts mainly as a transcription repressor [40], [41]. Our *in vivo* binding analysis suggested that AR binds the genomic sequence upstream to the transcription start site of coup-tf1 in several locations and the 5′ untranslated region (UTR) of the *coup-tf1* gene. In order to study the regulation of COUP-TF1 by AR we first further validated binding of the AR to the genomic sequences surrounding the coup-tf1 transcription start site. In the presence of the AR antagonist flutamide the AR is bound in the area 1–2 KB upstream to the TSS, in the coup-tf1 promoter and in the 5′ UTR of the gene. However in the presence of androgen the AR binds the area 1–2 kb upstream to the TSS, but not the promoter or 5′UTR (figure 2c and 2d).

As described above, COUP-TF1 mRNA levels are negatively regulated by androgen and positively regulated by flutamide. We further validated this observation at the protein level by western blot analysis. COUP-TF1 protein levels in LAPC4 cells do not change following addition of R1881 for 48 hours to androgen deprived cells. However, in the presence of flutamide for 48 hours, COUP-TF1 protein levels rise (figure 2b). Interestingly, concomitantly to upregulation of COUP-TF1, AR expression is downregulated by the addition of flutamide, demonstrating a negative feedback loop of AR activation in LAPC4 cells. In conclusion, as evidenced by quantitative real-time PCR and western blot analyses, antagonist bound AR positively regulates COUP-TF1 in LAPC4 cells.

### COUP-TF1 is expressed in malignant prostate epithelial cells and not in normal prostate epithelium

**Figure 3:**
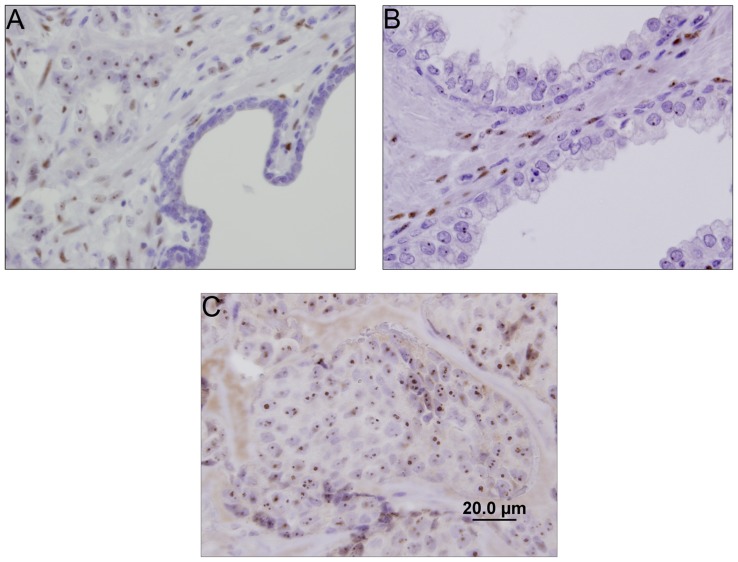
Immunohistochemical staining for COUP-TF1 in human prostate cancer, high grade PIN and benign epithelium. A. Immunohistochemical staining of human prostate cancer samples and adjacent benign glands for COUP-TF1 shows a nucleolar distribution of COUP-TF1 in malignant cells (upper left) and no COUP-TF1 staining in the adjacent benign gland (lower right). Stromal cells show nuclear staining of COUP-TF1. A representative of 28 samples analyzed is shown. B. Prostatic Intraepithelial Neoplasia (PIN) shows nucleolar distribution of COUP-TF1 and adjacent stromal cells show nuclear staining. C. immunostaining of LAPC4 xenografts shows nucleolar COUP-TF1 staining.

To examine the expression pattern of COUP-TF1 in human prostate and prostate cancer we used immunohistochemistry to detect COUP-TF1 in 28 human tumor samples. We detected COUP-TF1 in the malignant epithelium of 21 out of 28 primary prostate cancer samples examined. We could not find a correlation between COUP-TF1 staining and Gleason score or disease recurrence in either the epithelial or stromal cells. However, we did find significantly higher levels of COUP-TF1 staining in neoplastic prostate epithelium and in the pre-malignant prostatic intraepithelial neoplasia (PIN) compared with staining of adjacent benign prostate epithelium (figure 3a and 3b). This suggests that COUP-TF1 could play a role in early stages of prostate tumorigenesis. Interestingly, COUP-TF1 was distributed in epithelial cells in a nucleolar distribution, while stromal cells surrounding the epithelial neoplasia showed a nuclear pattern of staining.

In order to confirm COUP-TF1 antibody specificity we have stained LAPC4 xenografts for COUP-TF1 and found a nucleolar distribution of COUP-TF1 (figure 3c) similar to the distribution shown in human prostate cancer epithelium. Western blot analysis of those cells with the same COUP-TF1 antibody revealed a single band of 46 KD, corresponding to COUP-TF1.

**Figure 4:**
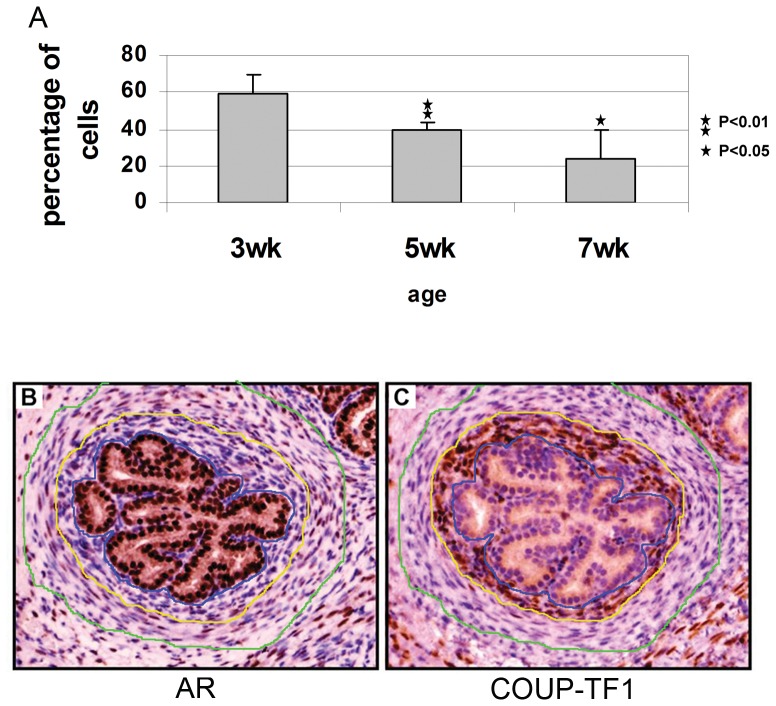
COUP-TF1 expression in the prostate of pre-pubertal mice. Prostates from pre pubertal Balb/c mice at ages 3, 5, 7 weeks were obtained and an immunohistochemical stain for COUP-TF1 was preformed. For each age group at least 4 mice were analyzed. A. stromal cell staining for COUP-TF1 was quantified by a pathologist as percent of stromal cells stained. An average of all lobes was calculated for each mouse and used for further analysis. P-value was calculated using student's t-test compared to age 3 weeks. B. A normal 3 week old mouse prostate was stained for AR and c. COUP-TF1. Serial sections from the same gland are shown. Epithelial cells are positive for AR and negative for COUP-TF1. Peri-epithelial stromal cells, between blue and yellow lines, are negative for AR and positive for COUP-TF1. Stromal cells more distant from the gland, between yellow and green lines, are positive for AR and negative for COUP-TF1. Similar inversely correlated staining patterns were seen in all slides examined.

In order to validate the negative regulation between AR and COUP-TF1 in the prostate we used pre-pubertal Balb/c mice. Pre-pubertal mice have a low testosterone level at three weeks, with levels increasing as the mouse reaches puberty [42]–[44]. We examined COUP-TF1 levels in the prostates of mice at ages 3, 5 and 7 weeks. Consistent with our observations in human samples, there was no COUP-TF1 staining in normal mouse prostate epithelium. However, we were specifically interested in COUP-TF1 levels in the prostate stroma (uro-genital mesenchyme), because of the well recognized crucial role of stromal AR in prostate development [45], [46]. Stromal-cell COUP-TF1 levels decreased with mouse age (figure 4a). Specifically, we could see a negative correlation between AR and COUP-TF1 levels in single prostate ducts in all slides examined (compare figure 4b and 4c). These findings show an inverse relationship between AR activation and COUP-TF1 expression in normal prostate development.

## Discussion

The transition of prostate cancer to the hormone refractory state is a major turning point in the progression of prostate cancer, and AR plays a major role in this transition. Currently used AR antagonists such as flutamide and bicalutamide, assume the role of receptor agonists when the disease becomes hormone refractory. The first evidence of this transition from antagonist to agonist was inferred from the clinical observation that prostate cancer patients had a 30% meaningful response to the withdrawal of a steroid hormone antagonist as the first maneuver after primary hormonal therapy failure [47]. This phenomenon prompted scientists to explore how AR antagonist can act as agonists in hormone refractory prostate cancer. Two studies showed that in HRPC, AR antagonists recruit coactivators (instead of co-repressors) to the AR bound target genes PSA and KLK2 giving one mechanistic explanation for this phenomenon [19], [20]. We hypothesized that in addition to this change taking place at the single target gene level, there is a global change in AR target genes which could add another explanation for the clinical observation. To this end, we first wished to compare in vivo DNA binding of AR bound to either flutamide or androgen.

The binding of AR to DNA, although ligand dependent, is not dependent on co-activators. Upon induction with flutamide, AR binds to the PSA promoter. The PSA promoter was also shown in different experiments to be bound by AR without any ligand (figure S2a), while the enhancer does not bind unliganded AR [28]. This prompted us to define the spectrum of ARBs between unliganded AR, agonist bound AR (R1881) and antagonist liganded AR (flutamide).

In our genome-wide location analysis of AR target genes we have discovered promoters that are bound constitutively with agonistic ligand, antagonist ligand or no ligand at all (figure S2). None of the few genes that were thought to be bound only in the presence of vehicle according to our *in vivo* binding analysis, were validated in gene specific chromatin immunoprecipitation analysis; thus it is less likely that ligand binding induces AR dissociation from chromatin.

Several genes were differentially bound by AR, in the presence of the agonist or antagonist (figure 2c and figure S2d). In order to asses the effect of AR binding on transcription we measured the expression of several of these ARB genes under the 3 different treatment conditions. Some of the constitutively bound genes were androgen regulated (e.g. STX6 and PSA). Others were expressed in LAPC4 but not regulated by androgen under the conditions in which they bind. Therefore, AR binding is not sufficient for transcriptional activation of ARBs or for androgen regulation. It is likely that additional conditions are required such as recruitment of co-activators, additional transcription factors or histone modifications. Alternatively, AR could be responsible for transcription initiation, but not for transcription elongation which is required for actively transcribed genes [48].

This differential binding in the presence of flutamide can be of special importance when considering antagonist to agonist conversion in hormone refractory prostate cancer. It is reasonable to assume that those genes can be easily induced upon transition to hormone refractoriness since they are already bound by an AR, and need only to further recruit co-activators and the basal transcription machinery. This finding could also have therapeutic significance utilizing the concept of synthetic lethality: Those target genes that are only activated in flutamide treated cells could serve as therapeutic targets in combination with flutamide.

In order to gain information on AR's activity in androgen dependent prostate cancer a bioinformatical analysis of AR target genes was performed. GO analysis of ARBs suggests that AR exerts its cellular effect by binding to promoters of other transcription factors in the presence of all the various ligands (table 1). This suggests that AR is a master regulator of prostate epithelial cells.

Next we looked for known and *de novo* motifs in ARBs. This analysis could identify transcription factors that act as co-regulators of transcription together with the AR. Our analysis revealed a non-consensus OCT1 motif that was reported previously to be involved in AR transcription [49]. Analysis of all known TRANSFAC motifs revealed both Brachyury and SOX9 motifs to be highly enriched in ARBs of all groups (table 2).

Brachyuri is a member of the T-box protein family that is extensively involved in embryogenesis [50], and although it was reported to be expressed in the prostate in large scale expression analyses [51], and in several prostate cancer cell lines [52], its precise role was never reported.

SOX9, is a Sry-related High Mobility Group (HMG) factor that was previously reported to play a role in prostate development. It is expressed at developing prostate epithelial buds with strongest expression in the distal tips of the buds. A severe defect in the development of the ventral prostate was observed in SOX9 mutant animals [53]. The possible role of SOX9 as an AR co–regulator needs to be further evaluated. However, the cooperative interaction of POU homeodomain proteins such as Oct1 or Oct4 with HMG factors such as SOX9 or SOX2 was previously described [54], [55]. This interaction is thought to be a fundamental mechanism of developmental control of gene expression. A core transcriptional regulatory circuit comprising OCT4, SOX2 and NANOG was described as controlling transcription of most developmentally important target genes in mouse embryonic stem cells [56]. Such cores are defined by identifying shared targets of key transcriptional regulators. A similar circuit was found in hepatocytes [57]. The results of this work raise the possibility that AR, OCT1 and SOX9 could be another module of co-operating transcription factors in the prostate.

Of further interest is the finding that AR preferentially binds AT-rich genomic areas. The biological significance of such binding should be further validated. This preferential AR binding to AT rich DNA could be explained by the relative lack of nucleosomes in AT rich regions, and specifically in areas containing poly (dA;dT) sites [58]. Areas that lack nucleosomes are more accessible to transcription factor binding, and in yeast, many transcription factors, although not all, prefer binding to areas of chromatin that lack nucleosomes [58]. Genomic areas that form boundaries that serve as nucleosome disfavoring areas are characterized by poly(dA∶dT) sites. Such sites are frequent in the AT rich region of ARBs. Yet if this was the case, one could expect to see such preferential binding with all transcription factors and this is not the case for at least one example. Recently He et al have shown in LNCAP prostate cancer cells that DHT treatment leads to depletion of nucleosomes from AR bound enhancers. The nucleosomes shift to areas flanking the AR binding sites. Analysis of the sequence between two flanking loci shows AT enrichment [59]. Another possibility is that the AR can recognize the specific conformational change that characterizes AT rich DNA [60], rendering recognition elements that are embedded in AT rich regions, more likely to be bound by AR.

In the second part of our work we wished to focus on AR regulation of one promoter, COUP-TF1. We chose COUP-TF1 for two reasons. First, because COUP-TF1 is a promoter that is occupied only by flutamide bound AR and not by androgen bound AR. It is important to show that such promoter occupancy serves a functional role. Second, as transcriptional repression by the AR is less well studied, we chose to further evaluate one novel AR target gene – COUP-TF1. COUP-TF1 is itself a transcription repressor known to play a role in development [61]–[65]. In breast cancer cell lines it was shown to enhance cancer proliferation and invasiveness [66].

We have demonstrated that COUP-TF1 is negatively regulated by AR in LAPC4 cells, both at the mRNA and protein levels. COUP-TF1 is down regulated upon treatment by the androgen R1881 and upregulated by treatment with flutamide.

We further wished to define the expression pattern of COUP-TF1 in prostate cancer. Immunohistochemical staining of COUP-TF1 in human prostate tumor tissue from radical prostatectomy specimens of various Gleason grades, and the adjacent normal epithelium showed higher staining for COUP-TF1 in nucleoli of malignant prostate epithelium compared to adjacent normal epithelium (figure 3a). This staining did not correlate with either Gleason score or recurrence of tumor after radical prostatectomy. The biologic role of nucleolar localization of a transcription factor is unclear. A proteomic analysis of nucleoli revealed the presence of 30 different transcription factors in this nuclear subcompartment [67]. COUP-TF1 was not found in that analysis of the nucleolar proteome, probably due to tissue specificity. However, the orphan nuclear receptor NR2E1, another member of the nuclear receptor subfamily 2, was found in the nucleolar proteome [68]. It was shown that nucleolar localization of transcription factors acts as a method of sequestration, and thus inhibition of activity [69], [70]. It would be interesting to further see whether COUP-TF1 is sequestered to nucleoli or sequesters other transcription factors to the nucleoli, thereby acting as a transcriptional repressor.

The important role of AR in prostate mesenchyme, and the role of AR in regulation of COUP-TF1 have led us to investigate the expression of COUP-TF1 in prostate mesenchyme. We found that COUP-TF1 levels in prostate mesenchyme are inversely correlated with androgen levels in prepubertal mice (figure 4a and 4b) in agreement with its possible down regulation by androgens.

The results presented in this work further promote our understanding of the importance of AR antagonist bound target genes, which are expected to play a major role in HRPC, and are possibly targets for therapy. These results, specifically with their bioinformatic analysis, provide a basis for further study on AR's role and mechanism of activity. We explored one AR target and analyzed its expression in both malignant and normal tissue. Other targets revealed in this work can be explored in a similar way, in order to investigate their role in androgen dependent and hormone refractory prostate cancer. The AR co-activators we suggested, and the AT rich environment of AR binding, should be further evaluated in biological experiments to validate their role and significance in prostate cancer.

## Materials and Methods

### Ethics statement

Experiments with human tissues were approved by the Institutional Review Board (IRB), at the Hadassah-Hebrew University Medical Center. Due to the retrospective nature of this study, the fact that tissues were unidentified and according to the declaration of Helsinki, our IRB waived the need for written informed consent. IRB approval number HMO0416-08. All mice experiments were approved by the Hadassah-Hebrew University Medical Center Institutional Animal Care and Use Committee - approval number MD 78.06-3.

### Cell lines

The LAPC4 cell line was generated from an androgen dependent human prostate cancer [22]. The cell line was grown in RPMI 1640 (Biological Industry, Beit-Haemek, Israel) supplemented with 10% Fetal Bovine Serum (Biological Industry, Beit-Haemek, Israel) and 10 nM R1881 (Perkin Elmer, Waltham, MA, US).

### Cell proliferation assay

LAPC4 cells were cultured in 96 well plates, 5×10^3^ cells per well. All treatments were performed on the same plate, and each treatment was repeated in 8 wells. Cells were plated in RPMI 1640 without phenol red supplemented with charcoal striped serum (CSS) with either ethanol (as vehicle), DHT 10 nM, flutamide 40 µM or the combination of DHT and flutamide (Sigma Chemicals, St. Louis, MO, US). Seven days after plating XTT reagent (Biological industries, Beit Haemek, Israel) was added to the plate and absorbance was determined at 460 nm.

Absorbance of wells containing medium alone was subtracted, and fold change compared to vehicle was calculated, as an average of 8 replicates. P value was calculated using student's t-test compared either to vehicle (for DHT and flutamide treatment groups) or to DHT treatment (for the combined treatment group).

### Chromatin Immunoprecipitation (ChIP)

A total of 5×10^7^ LAPC4 cells were used for each reaction. Cells were grown in RPMI 1640 without phenol red with CSS for three days. The media was then supplemented with ethanol (as vehicle), R1881 10 nM or flutamide 40 µM. The cells were fixed with 1% Formaldehyde for 10 minutes. Then cells were lysed in lysis buffer containing 50 mM Tris pH 8, 1% SDS, 10 mM EDTA and Protease inhibitor cocktail (Sigma Chemicals, St. Louis, MO, US) and sonicated to yield fragments of 200–500 bp. 1/30 of the input sample was taken for further standardization. Lysates were pre-incubated with 60 µl salmon sperm coated protein G beads (Upstate Biotechnology, Lake Placid, NY, US) in 50% slurry. Than lysates were incubated with 10 µg of monoclonal anti-AR antibody clone AR441 (Santa Cruz Biotechnology, Santa Cruz, CA, US) over-night. 60 µg Salmon sperm protein G beads were added for 1 hour. Beads were than washed twice with 1 ml of each of three wash buffers as recommended by upstate biotechnology ChIP protocol (Low salt wash buffer: 0.1% SDS, 1% Triton X-100, 2 mM EDTA, 20 mM Tris-HCl pH 8, 150 mM NaCl; High Salt wash buffer: 0.1% SDS, 1% Triton X-100, 2 mM EDTA, 20 mM Tris-HCl pH 8, 500 mM NaCl; LiCl buffer: 0.25 M LiCl, 1% NP40, 1% deoxycholate, 1 mM EDTA, 10 mM Tris HCl pH 8). Elution was done using TE buffer (10 mM Tris pH 8.0, 1 mM EDTA) and 1% SDS for 30 minutes. The immunoprecipitated and input samples were reverse cross linked by incubation at 65°C for 4–16 hours and then proteinase K was added for purification. The eluted and input samples were purified using Qiaquick PCR purification kit (Qiagen, Hilden, Germany). A sample of input was run on agarose gel to ensure DNA fragmentation to 200–500 bp fragments and quantified using NanoDrop to about 400 ng/µl. The IP sample had a concentration of approximately 10 ng/µl.

### Semi quantitative PCR for Chromatin IP

Quantification of ChIP was done using a standard gene, either histone H2A promoter or GAPDH promoter. The input sample was diluted 1∶300 followed by four consecutive three-fold dilutions. IP was used as template in 0.06 µl and 0.18 µl per reaction. PCR for promoter of interest and reference gene were done using Abgene Reddymix (ABgene, Surrey, UK) with annealing temperature of 56–60°C, 37 cycles. A sample of PCR product was resolved on a 1.5% Agarose- Ethidium Bromide gel, and documented using GelDoc-It imaging system.

We wrote a Matlab (Mathworks, Natick, MA, US) program to quantify specific promoter enrichment compared to a standard gene, in a non-biased way. Input samples were set as the standard and piecewise linear interpolation was done. IP samples were quantified according to that interpolation. Enrichment of the promoter in hand was calculated as specific promoter on standard divided by reference gene on standard. An average of two IP samples was used. Each PCR was repeated at least twice, and represents at least two independent ChIP reactions. For semi-quantitative PCR primers see table S1.

### Genome-wide location analysis (ChIP on chip)

Protocol for ChIP amplification and hybridization was done as previously described [71]. In brief, ChIP was done as described above. DNA of input sample and IP were blunted using T4 DNA Polymerase (New England Biolabs, Ipswich, MA, USA) and amplified using ligation-mediated PCR (primers oJW102 5′ -GCGGTGACCCGGGAGATCTGAATTC-3′, oJW103 5′- GAATTCAGATC-3′ [71]). After ligation and amplification the samples were labeled using Klenow fragment (New England Biolabs, Ipswich, MA, USA). Input and IP were labeled with different labels (Cy3/Cy5-dCTP), and IP and input labels were switched between samples. Labeling and DNA quantity was measured using NanoDrop. Samples were used only if DNA concentration was above 50 ng/µl and cy3/cy5 concentration was above100 pmol/µl.

Input and IP were purified using Qiaquick PCR purification kit (Qiagen, Hilden, Germany), mixed and hybridized to HU19K spotted promoter array [37]. Yeast tRNA (Invitrogen, Carlsbad, CA, USA) and human cot-1 DNA (Invitrogen, Carlsbad, CA, US) were used for hybridization blockage.

The array was scanned and analyzed with GenePix Pro software (Molecular Devices, Sunnyvale, CA, US), and the fluorescence intensity in both channels was obtained for each spot.

### Hu19K microarray

The Hu19K array was manufactured and kindly provided by Richard A. Young's lab [37]. This array is an extension of a former array, Hu13K, which was generated in the same lab [72]. Detailed explanation of array construction can be found in [37]. In brief, Hu19K is a custom made spotted array, designed to analyze binding to promoter sequence, i.e. −750 bp to +250 bp in relation to the TSS. In addition it contains tiling of the area −3375 bp to +2375 bp relative to the TSS of 276 diverse genes. A total of 175 micro RNA loci containing regions were included. There were 623 probes designed for areas >800 kb from the nearest gene, and these were named intergenic areas.

### AR binding site analysis

For each probe the intensity was normalized according to whole chip error model which incorporates signal intensity and background noise to calculate p-value for the significance of enrichment for each probe. A probe with enrichment p<0.001 was considered as a bound promoter. A probe that was enriched p<0.001 in one array and p<0.01 in another was considered to be enriched in both arrays.

### DNA motif search

The bound probes were analyzed with 300 bp added on each side to compensate for length of sonicated genomic DNA fragments (200–500 bp). We then applied a series of motif search algorithms. To search for well defined motifs, without enumeration (such as the ARE, ARE half site and canonical and non-canonical OCT1 elements), we used the motif search procedure FindPatterns from the GCG package (GCG Version 11.1, Accelrys Inc., San Diego, CA, US). Motifs of 6 bp to 8 bp length were searched with no mismatches allowed, and 9 to12 bp sequences were searched with two mismatches allowed. P-value of the number of hits, compared to the array's background, was calculated using corrected hypergeometric distribution.

We further performed Position Weight Matrix (PWM) based motif scanning from the TRANSFAC database, release 12.1 [73] using CIS [32]. The significance of each motif was calculated by comparing the number of times the motif was found in each of the ARB lists compared to the number of times it was found in the array's non-bound promoters. P-values were calculated using a hyper-geometric enrichment test. The threshold for similarity between TRANSFAC motif and probe was set so 5% of non-bound probes are positive for motif. Therefore, any motif that appears in more then 5% of bound promoters is enriched, with a p-value as stated before.

*De novo* motif search was done using Weeder [36], and the sequences found were searched in the other groups using FindPattern as described above.

### GO analysis

Gene Ontology (GO) category analysis was performed using Webgestalt software [31]. Number of genes in each category was calculated. Expected number in each category was calculated based on number of genes in each dataset and in each category. Then p-value was calculated using hypergeometric test and was adjusted using multiple test adjustment. P-Values<0.01 are shown.

### RNA extraction and cDNA preparation

For expression assays cells were grown in RPMI 1640 without phenol red with CSS for three days. The media was then supplemented with ethanol (as vehicle), R1881 10 nM or flutamide 40 µM for 24 hours. RNA was extracted from LAPC4 cells after various treatments using Trizol reagent (Sigma Chemicals, St. Louis, MO, USA), as stated in the manufacturer protocol. RNA was separated using chloroform, and purified using isopropanol and ethanol. The RNA was purified again using ethanol precipitation, and quantified using NanoDrop.

Reverse transcription PCR was preformed using High capacity cDNA Reverse Transcription kit (Applied Biosystems, Carlsbad, CA, USA).

### Quantitative real time PCR

Real time PCR was performed using Platinum SYBR green qPCR SuperMix-UDG with Rox (Invitrogen, Carlsbad, CA, US), according to the manufacturer's instructions in an Applied Biosystems 7900HT real time PCR machine (Applied Biosystems, Carlsbad, CA, US). See primers used in table S1.

### Co-immunoprecipitation

Co-immunoprecipitation was preformed according to [28]. Briefly, LAPC4 cells were cultured in 10 cm plates. 10^7^ cells were harvested for each reaction. Cells were lysed in Buffer A (50 mM Tris-Cl pH 7.4, 150 mM NaCl,5 mM EDTA, 0.5% Nonidet P-40, protease inhibitor cocktail). 1% was taken for input sample.

Lysate was precleared with protein A sepharose beads and then either anti AR (AR441, Santa Cruz Biotechnology, Santa Cruz, CA, US) or control Ab (anti Lyn, H-6, Santa Cruz Biotechnology, Santa Cruz, CA, US) were added overnight. Than samples were incubated with sepharose A beads for one hour and washed with buffer A three times. Samples were analyzed on SDS-PAGE gel. Western blot was preformed as described below using antibodies recognizing AR (AR441, Santa Cruz Biotechnology, Santa Cruz, CA, US) or SOX9 (Millipore, cat #AB5535, Temecula, CA, US).

### Western blot analysis

For protein expression assays cells were grown in RPMI 1640 without phenol red supplemented with CSS for three days. The media was then supplemented with ethanol (as vehicle), R1881 10 nM or flutamide 40 µM for 48 hours. Proteins were extracted using lysis buffer containing 50 mM Tris pH 7.6, 150 mM NaCl, 0.5% NP-40, 5 mM EDTA, protease inhibitor cocktail 1∶100. Protein concentrations were determined using Bradford assay, and 50 µg of total protein was loaded on 9% SDS-PAGE gel. Proteins were transferred to a nitrocellulose membrane and were blotted with polyclonal rabbit anti-AR (sc-816, Santa Cruz, CA, USA) at 1∶1000 dilution, mouse monoclonal anti-COUP-TF1 (H8132, Abcam, Cambridge, UK) at 1∶1000 dilution and mouse monoclonal anti-GAPDH (clone 6C5, Chemicon International, Millipore, Billerica, MA) at 1∶30,000 dilution, all for one hour at room temperature. Secondary antibodies used were HRP conjugated goat anti-mouse or anti-rabbit (Jackson ImmunoResearch laboratories, Philadelphia, USA) diluted 1∶10000 for 45 minutes at room temperature. ECL reagents were purchased from Amersham Pharmacia, NJ, USA and used according to manufacturer's recommendations.

### Human prostate cancer samples

The study included 28 samples of human prostate carcinoma from radical prostatectomy samples from patients previously untreated. The Gleason score was obtained from the pathology report, and information regarding recurrence of disease after prostatectomy was obtained from the patients' records.

### Mice prostate samples

Murine prostates were taken from Balb/c mice at defined ages. At least four mice in each group were used. Anterior, ventral and dorsolateral prostate samples were obtained.

### Xenografts

LAPC4 cells were harvested, washed and reconstituted in PBS. 10^6^ cells were injected subcutaneously to 6–7 weeks old nude male mice together with Matrigel (BD biosciences, Bedford, MA, US). A total of 4 mice were injected. Tumors were harvested when reached measurable size and formalin fixed.

### Immunohistochemistry

Four micrometer thick sections of paraffin embedded human or mouse tissue were de-paraffinized in xylene and rehydrated in graded alcohols. Endogenous peroxidase was blocked using 3% hydrogen peroxide for 5 minutes before and after antigen retrieval. Antigen retrieval was preformed using 50 mM EDTA pH 8.0 in a pressure cooker for 3 minutes (AR) or using 100 mM Glycine pH 9.0 in a pressure cooker for 3 minutes, twice (COUP-TF1). Primary antibodies were used as follows: polyclonal rabbit anti-AR (sc-816, Santa Cruz, CA, US) at 1∶500 dilution and mouse monoclonal anti- COUP-TF1 (H8132, Abcam, Cambridge, UK) at 1∶150 dilution. Both were diluted in Cas block (Invitrogen, Carlsbad, CA, US) and incubated with target tissues at room temperature for one hour. Detection was done using Mach2 and Mach3 AP polymer detection kits (Biocare Medical, Concord, CA, US). Staining was done using DAB for two minutes and counter staining using hematoxylin for 30 seconds. Staining was quantified as percent of cells stained, by a pathologist (EP) who was unaware of the experimental group.

## Supporting Information

Figure S1 **Effect of androgen modulation on growth of LAPC4 cells in vitro.** LAPC4 cells were grown in the presence of vehicle, androgen (DHT 10 nM), the AR antagonist flutamide (40 µM) or the combination of androgen and flutamide. Cell growth was monitored on day 7 compared to vehicle using XTT assay. Each datapoint represents the average of 8 independent wells. Y axis – fold proliferation compared to vehicle. P-value was calculated using student's t-test compared either to vehicle (for DHT and flutamide treatment groups) or to DHT treatment (for the combined treatment group).
(TIFF)

Figure S2 **Validation of novel AR target genes.** LACP4 cells were androgen deprived for 72 hours and then treated with vehicle (ethanol), a synthetic androgen (R1881) or an AR antagonist (flutamide). Cells were fixed 16 hours after treatment and chromatin immunoprecipitation with an anti AR antibody was performed. PCR for the indicated target genes compared to non-bound gene are presented for 3 fold dilutions of input and immunoprecipitated fraction. Enrichment of each promoter compared to a non-bound promoter is quantified below each image using a Matlab procedure designed to calculate enrichment in an unbiased manner. Each experiment represents at least two different chromatin IP and at least two PCR reactions for each chromatin IP. a. PSA b. SOX5 c. DOCK1 d. B3GNT5 e. SLITRK3 f. IL1R2.
(TIF)

Figure S3 **Chromosomal distribution of AR target genes.** Chromosomal distribution of AR target genes in the three treatment groups. Red dots indicate chromosomal locations of AR bound promoters.
(TIF)

Table S1 **Primers used for PCR reactions.**
(PDF)

Table S2 **Genomic locations (Hg19) and p value of enrichment upon treatment with different ligands.**
(XLS)
