# Supplementary figures and images for: Genome-Wide Analysis of Androgen Receptor Targets Reveals COUP-TF1 as a Novel Player in Human Prostate Cancer
Source: PLoS One. 2012 Oct 4;7(10):e46467. doi: 10.1371/journal.pone.0046467 (PMC3464259; doi:10.1371/journal.pone.0046467)

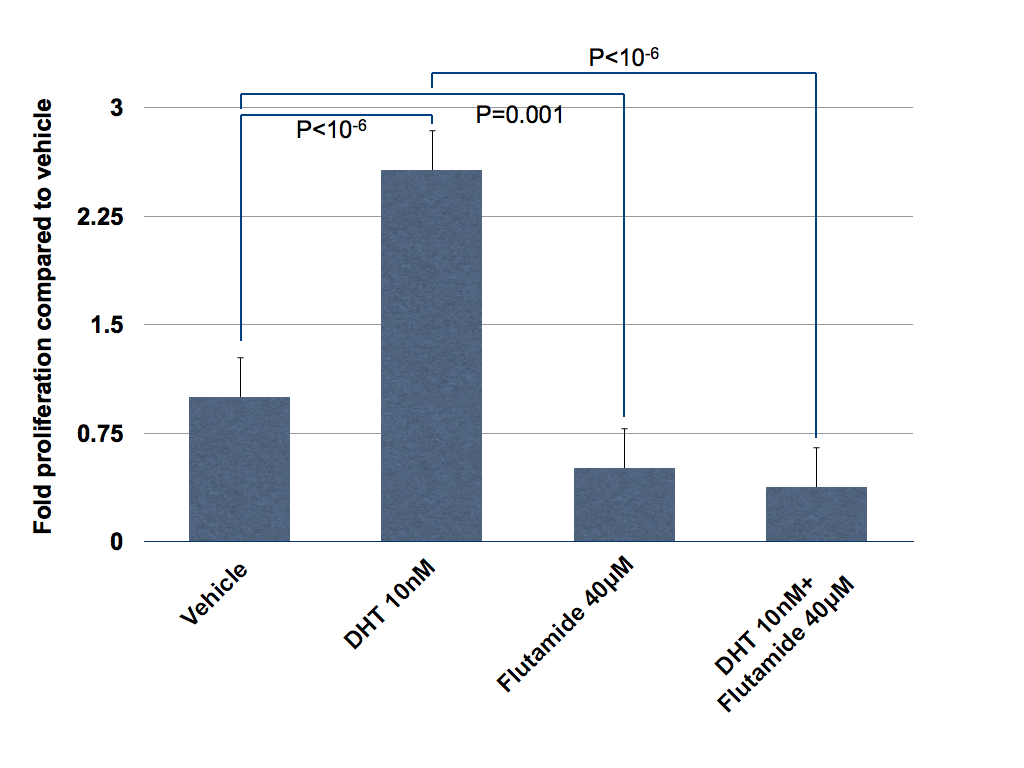

Supplement: Figure S1 — Effect of androgen modulation on growth of LAPC4 cells in vitro. LAPC4 cells were grown in the presence of vehicle, androgen (DHT 10 nM), the AR antagonist flutamide (40 µM) or the combination of androgen and flutamide. Cell growth was monitored on day 7 compared to vehicle using XTT assay. Each datapoint represents the average of 8 independent wells. Y axis – fold proliferation compared to vehicle. P-value was calculated using student's t-test compared either to vehicle (for DHT and flutamide treatment groups) or to DHT treatment (for the combined treatment group). (TIFF) [file pone.0046467.s001.tiff]

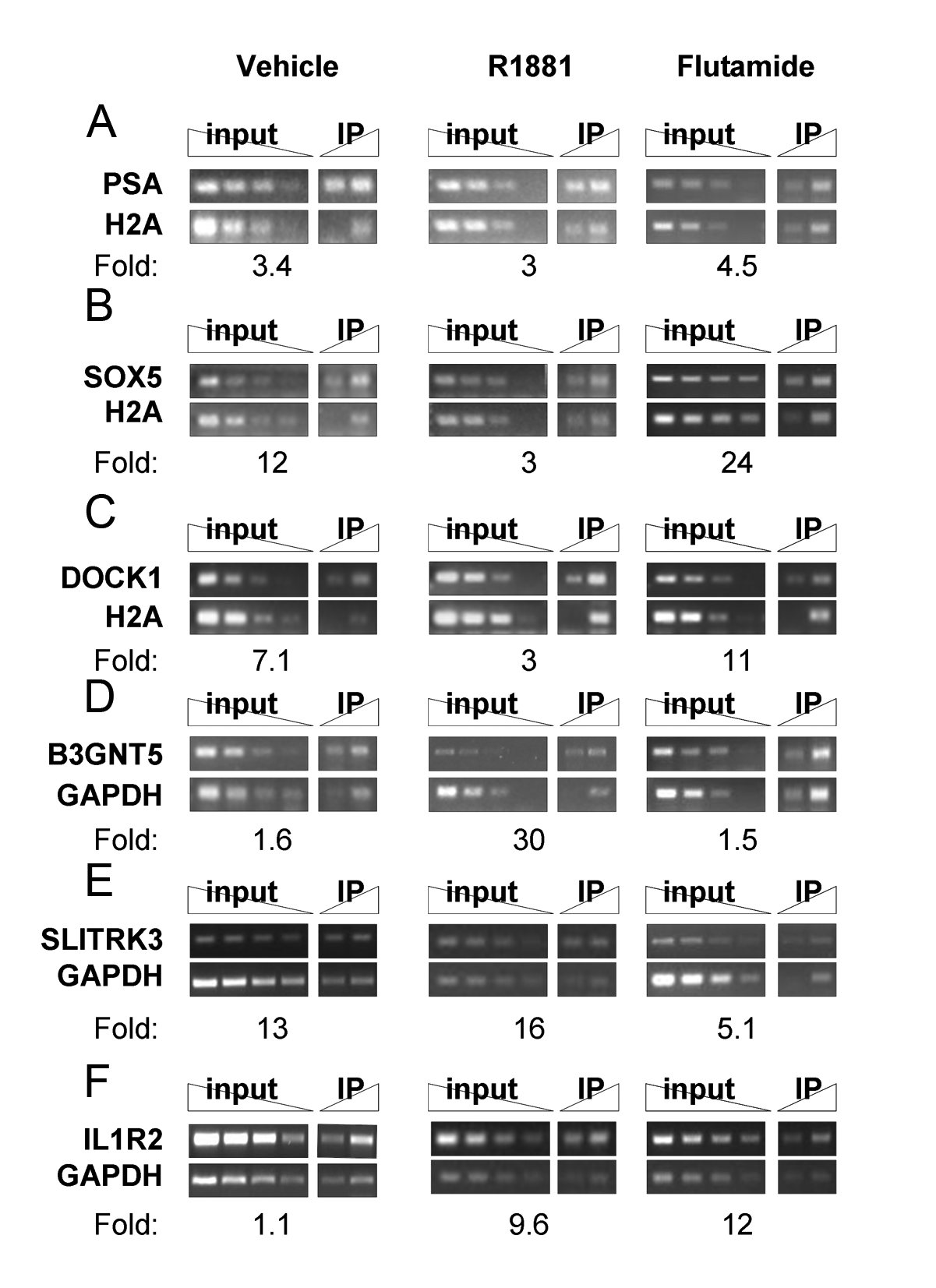

Supplement: Figure S2 — Validation of novel AR target genes. LACP4 cells were androgen deprived for 72 hours and then treated with vehicle (ethanol), a synthetic androgen (R1881) or an AR antagonist (flutamide). Cells were fixed 16 hours after treatment and chromatin immunoprecipitation with an anti AR antibody was performed. PCR for the indicated target genes compared to non-bound gene are presented for 3 fold dilutions of input and immunoprecipitated fraction. Enrichment of each promoter compared to a non-bound promoter is quantified below each image using a Matlab procedure designed to calculate enrichment in an unbiased manner. Each experiment represents at least two different chromatin IP and at least two PCR reactions for each chromatin IP. a. PSA b. SOX5 c. DOCK1 d. B3GNT5 e. SLITRK3 f. IL1R2. (TIF) [file pone.0046467.s002.tif]

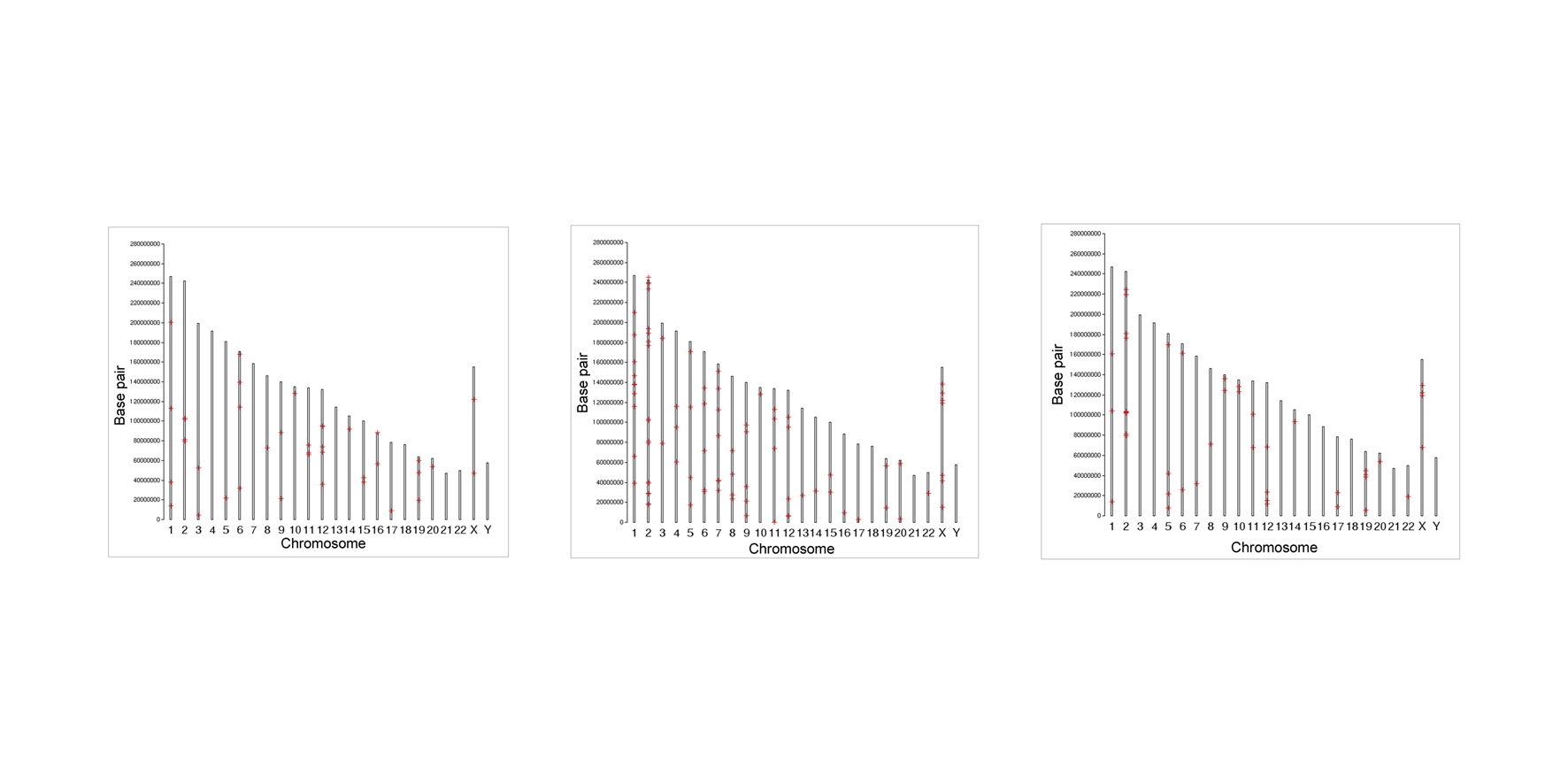

Supplement: Figure S3 — Chromosomal distribution of AR target genes. Chromosomal distribution of AR target genes in the three treatment groups. Red dots indicate chromosomal locations of AR bound promoters. (TIF) [file pone.0046467.s003.tif]
